# Outpatient healthcare access and utilization for neonatal abstinence syndrome children: A systematic review

**DOI:** 10.1017/cts.2019.407

**Published:** 2019-08-29

**Authors:** Adam Van Horn, Whitney Powell, Ashley Wicker, Anthony D. Mahairas, Liza M. Creel, Matthew L. Bush

**Affiliations:** 1 Department of Otolaryngology – Head and Neck Surgery, University of Kentucky Medical Center, Lexington, KY, USA; 2 University of Kentucky College of Medicine, Lexington, KY, USA; 3 Department of Health Management and Systems Sciences, School of Public Health and Information Sciences, University of Louisville, Louisville, KY, USA

**Keywords:** Neonatal abstinence syndrome, healthcare access, healthcare utilization

## Abstract

**Objective:**

The objective of this study was to systematically assess the literature regarding postnatal healthcare utilization and barriers/facilitators of healthcare in neonatal abstinence syndrome (NAS) children.

**Methods:**

A systematic search was performed in PubMed, Cochrane Database of Systematic Reviews, PsychINFO, Cumulative Index to Nursing and Allied Health Literature (CINAHL), and Web of Science to identify peer-reviewed research. Eligible studies were peer-reviewed articles reporting on broad aspects of primary and specialty healthcare utilization and access in NAS children. Three investigators independently reviewed all articles and extracted data. Study bias was assessed using the Newcastle–Ottawa Assessment Scale and the National Institute of Health Study Quality Assessment Tool.

**Results:**

This review identified 14 articles that met criteria. NAS children have poorer outpatient appointment adherence and have a higher rate of being lost to follow-up. These children have overall poorer health indicated by a significantly higher risk of ER visits, hospital readmission, and early childhood mortality compared with non-NAS infants. Intensive multidisciplinary support provided through outpatient weaning programs facilitates healthcare utilization and could serve as a model that could be applied to other healthcare fields to improve the health among this population.

**Conclusions:**

This review investigated the difficulties in accessing outpatient care as well as the utilization of such care for NAS infants. NAS infants tend to have decreased access to and utilization of outpatient healthcare following hospital birth discharge. Outpatient weaning programs have proven to be effective; however, these programs require intensive resources and care coordination that has yet to be implemented into other healthcare areas for NAS children.

## Introduction

Neonatal abstinence syndrome (NAS) is a significant public health problem in the USA and abroad. There is an increasing incidence of NAS, leading to expanding costs to healthcare systems [1–3]. The majority of published literature on the subject focuses on prenatal prevention, postnatal diagnosis, and effective treatment strategies during the birth hospitalization to better identify and safely treat these neonates. Successful substance treatment and weaning strategies are critical issues with this vulnerable population during birth hospitalization and immediately following discharge; however, these infants are at risk of having other health conditions being dismissed or delayed in the outpatient setting. The complex interconnected medical problems that these children face requires multidisciplinary care. Careful outpatient coordination of care and discharge planning is a vital part of management; yet, there is a gap in the literature on factors that influence the delivery of outpatient care in this population [4]. While there is evidence of long-term complications of NAS a variety of health conditions, limited research has explored the barriers and facilitators to outpatient healthcare access and utilization for these children following discharge from the hospital [5].

The complex socioeconomic environment of many of these infants complicates the delivery of postnatal outpatient healthcare. Several studies have characterized the environment surrounding these children. In general, those findings have shown lower levels of parental education, lower socioeconomic status, and increased likelihood of being uninsured, having a break in insurance coverage, or being covered by public health insurance [6,7]. Infants diagnosed with NAS have increased rates of placement into foster care or adoption services and social situations characterized by parental abuse with or without involvement of child protective services [8–11]. Geographically, the incidence rates of NAS and maternal opioid use are significantly higher in rural areas than urban areas and the growth of these rates in rural regions is alarming [7,12]. These environmental factors could further complicate the access to timely preventative care as well as multidisciplinary specialty outpatient healthcare. The objective of this study was to systematically assess the literature regarding postnatal healthcare utilization and barriers of healthcare in NAS children following hospital birth discharge.

## Methods

This study was exempt from Institutional Review Board approval. The primary research objective was to answer the question: Do NAS infants have poorer utilization of healthcare (either primary or specialty) following discharge compared with non-NAS infants? The primary outcome for healthcare utilization was derived from data reporting hospital readmission, ER visits, and metrics regarding ambulatory specialty or primary care clinic utilization (appointment adherence or no-show). We also sought to describe patient-level sociodemographic and medical factors, as well as, healthcare system factors (either facilitators or barriers) that influence ambulatory healthcare utilization for NAS infants. Our review sought to assess a broad scope of medical disciplines focused on healthcare utilization following the birth hospitalization in NAS patients with specific reporting of access to care, barriers/facilitators to care, healthcare utilization, and patient follow-up. The Preferred Reporting Items for Systematic Reviews and Meta-Analysis checklist was used to guide our review [13]. The specific inclusion criteria included: (1) peer-reviewed articles reporting on broad aspects of healthcare utilization in NAS children, (2) articles reporting sociodemographic data of NAS children, and (3) articles with a pediatric population (< 18 years of age). Exclusion criteria included: (1) case reports or non-original research and (2) language other than English.

### Search Strategy

**Fig. 1.**
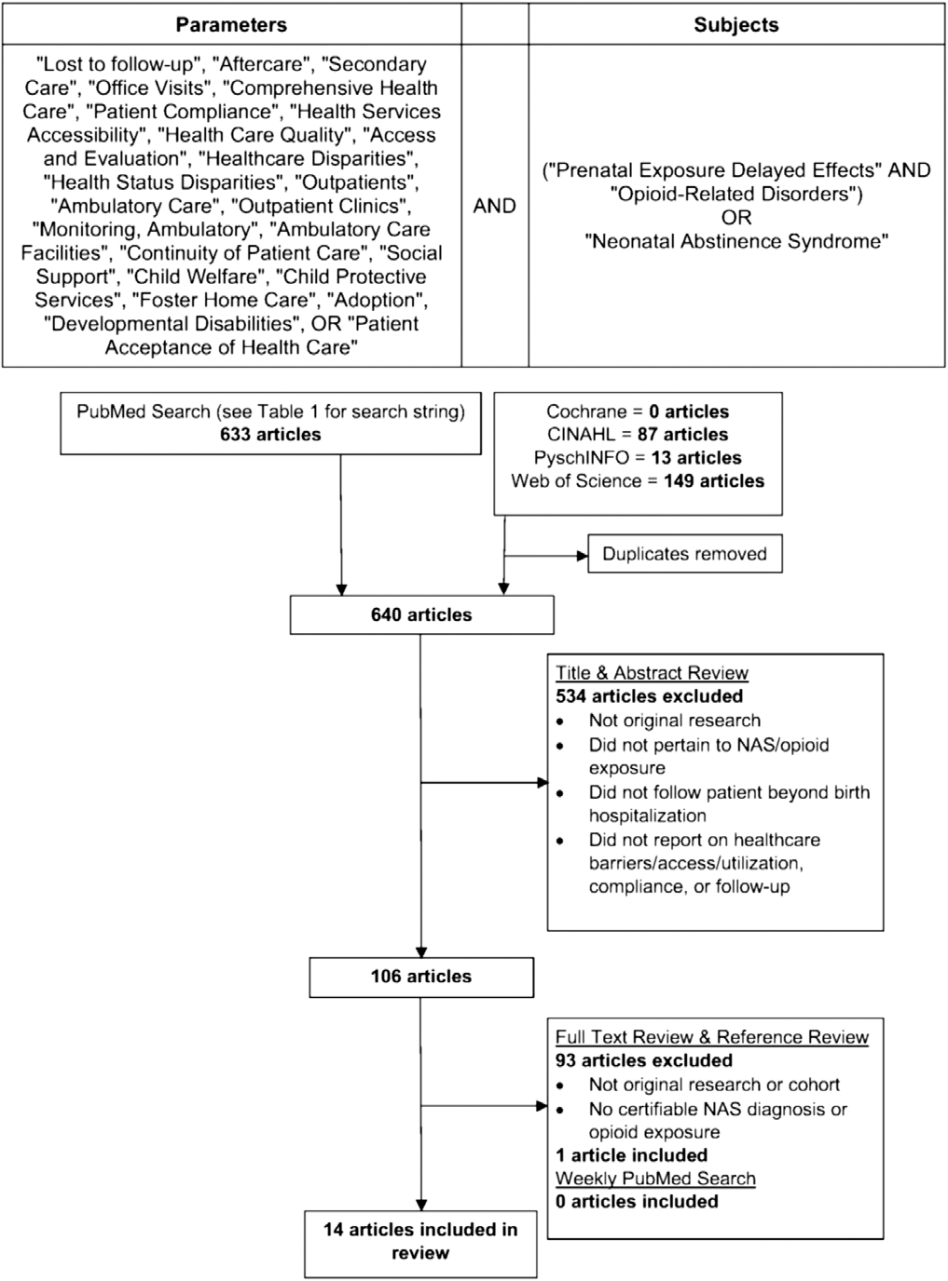
The systematic review search string and preferred reporting items for systematic review and meta-analysis (PRISMA) algorithm. CINAHL, Cumulative Index to Nursing and Allied Health Literature; NAS, neonatal abstinence syndrome.

To perform a systematic literature review of healthcare utilization in NAS children, a search string was developed to include a wide range of terms connected to healthcare utilization and access along with neonatal substance exposure terms such as prenatal exposure delayed effects, opioid-related disorders, and NAS. The National Library of Medicine’s medical subject headings (MeSH) were used to develop a search that could capture articles concerned with the aforementioned concepts as they related to NAS patients or neonates exposed to opioids *in utero*. The MeSH parameters used to identify articles of interest and the MeSH used to identify appropriate study subjects are listed in Fig. 1.

An initial search was performed on July 30, 2017 in PubMed using the search string in Table 1, employing [All Fields] so that the MeSH-indexed terms would be captured regardless of the term being indexed, in title, or body of article. Searches were limited to English articles with no date of publication limitations. Next, the same terms were used to conduct searches on the same date within Cochrane Database of Systematic Reviews (CDSR), Cumulative Index to Nursing and Allied Health Literature (CINAHL), PsychINFO, and Web of Science. Duplicate studies already found within the PubMed search were removed. The search string was repeated in PubMed weekly up to March 25, 2018 with studies not included in the original search being added for review. Additional articles were identified through review of the references section of the publications of the original search.

**Table 1.** Quality assessment of articles

(A) Cohort and case-control studies using the Newcastle–Ottawa Scale										
Non-randomized cohort studies										
Author (Year)	Selection				Comparability		Outcome			Total
	Cohort representative?	Selection of non-exposed cohort?	Assessment of exposure?	Outcome not present at onset?	Control for primary factor?	Control for secondary factor?	Assessment of outcome?	Long enough follow-up?	Adequacy of follow-up?	
Fang (2015) [25]	+	+	+	+	+		+	+	+	8
Witt (2017) [28]	+	+	+	+	+		+	+	+	8
Patrick [1]	+	+	+	+	+		+	+		7
Uebel (2015) [27]	+	+	+	+	+		+	+	+	8
Kelly (2015) [16]	+		+	+			+	+	+	6
Smirk (2014) [33]	+	+	+	+			+	+		6

Case-control studies										
Author (Year)	Selection				Comparability		Exposure			Total
	Case definition adequate?	Cases representative?	Selection of controls?	Definitions of controls?	Control for primary factor?	Control for secondary factor?	Assessment of exposure?	Same assessment for cases/controls?	Non-response rate?	
Kvisto (2014) [15]	+		+	+			+	+		5
Payot (2000) [17]	+	+	+	+			+	+		6
Backes (2012) [31]	+		+	+			+	+		5

(B) Case series studies using the National Institute of Health Study Quality Assessment Tool										
Case series studies										
Author (Year)		1	2	3	4	5	6	7	8	9
Gill (2003) [20]		+	+	+	+	+	+	+	+	+
Chau (2016) [29]		+	+	–	+	+	+	–	+	+
Dickes (2017) [32]		+	+	–	+	+	–	–	+	+
Lai (2017) [30]		+	+	–	+	+	+	+	+	+
Oei (2001) [19]		+	+	+	+	+	+	–	+	+

Categories for assessing bias in case series studies: (1) Research objectives stated? (2) Sample defined? (3) Were cases consecutive? (4) Subjects comparable? (5) Intervention clearly defined? (6) Were outcome measures clearly defined? (7) Length of follow-up adequate? (8) Statistical methods well described? (9) Results well described?.

### Study Selection

In screening the studies identified through this search strategy, we identified two major themes in the research in this population: (1) studies addressing utilization of care in variety of healthcare setting in NAS patients following their birth hospitalization, (2) studies addressing factors influencing adherence and utilization with outpatient weaning programs of NAS patients. Studies within both themes were included in this review. Articles, titles, and abstracts were reviewed independently by three reviewers who selected or removed them based on the inclusion and exclusion criteria of the review. Disagreements at this level of review were settled by majority consensus among the three reviewers. This then created a list of studies for the final review and data extraction. Additional studies from the automated weekly PubMed searches were reviewed for inclusion if appropriate as outlined above. Studies were included if the study described original research (prospective or retrospective) examining participants with NAS or *in utero* opioid exposure. No specific intervention was necessary as the questions addressed by our review were not concerned with outcomes from specific interventions. Studies had to include outcome measures assessing at least one of the following parameters in participants removed from their birth hospitalization: outpatient follow-up rates, outpatient treatment compliance rates, access and/or barriers to healthcare, or healthcare utilization. Studies were excluded if the research was not original, participants had no exposure to opioids *in utero*, outcome measures did not address the above criteria, or outcomes were limited to the birth hospitalizations.

### Data Extraction and Quality Assessment

The final list of studies was organized into the two themes described above. Data were collected on specific areas: access or barriers to care for NAS patients examined after birth hospitalization, healthcare utilization in the NAS patients after birth hospitalization discharge, NAS patient follow-up, treatment compliance and support mechanisms within outpatient NAS weaning programs. The data for those specific outcomes were recorded within templates constructed for each section. There was inconsistency of outcome measure reported, thus no meta-analysis was performed and only qualitative data synthesis was performed. The level of evidence of each article was assessed based on the standard Oxford Centre for Evidence-based Medicine guidelines [23]. The study quality/bias was determined using the Newcastle–Ottawa Scale (NOS) for case-control or cohort studies (Table 1) [24]. The quality/bias of case series was assessed using the National Institute of Health (National Heart, Lung, and Blood Institute) Study Quality Assessment Tool [14] (Table 1).

## Results

The PubMed search yielded 633 articles. Searches within the CDSR, CINAHL, PsychINFO, and Web of Science databases added 7 articles after 242 duplicates were removed. These 640 articles were included in the title and abstract review with 534 failing to meet inclusion criteria. The remaining 106 articles underwent a full text and reference review with 1 additional article being identified. Zero additional articles were identified in the weekly PubMed searches. Fourteen articles met our criteria and were included in the final systematic review. This process is summarized in Fig. 1. The 14 articles were divided into the 2 aforementioned themes. Seven articles addressed the general NAS population with characterization of healthcare utilization following the birth hospitalization. These studies also highlight some potential barriers to care within the study populations. Another seven articles focused on the results of outpatient weaning programs with factors related to ambulatory program adherence and follow-up rates.

### Healthcare Utilization Among NAS Infants

Seven studies included in the final review examined healthcare utilization following the birth hospitalization. These studies are summarized in Table 2 and characterize NAS infant healthcare utilization following birth hospitalization. Four of the seven studies utilized multivariate analysis to control for confounding maternal and child factors that could influence utilization. Fang et al found a lower likelihood of primary care preventative care visits (0.85 adjusted relative risk) and higher likelihood of ER visits (1.46 adjusted relative risk) in NAS infants after hospital discharge, compared to non-NAS infants. Furthermore, this study reported higher rates of breaks in insurance coverage for infants born to mothers with opiate abuse history compared to unexposed infants, regardless of the infant being born prior to or after the mother’s enrollment in a methadone maintenance program [25]. Patrick et al compared an NAS cohort, a late preterm cohort, and an uncomplicated term infant cohort and found that NAS infants had a significantly higher rate of Medicaid insurance and lower rate of private insurance coverage compared with the other groups [26]. This study also found that, compared to the other cohorts, NAS infants had higher hospital readmission rates. Payot et al found that infants of opioid-dependent mothers had lower usage of outpatient clinics, lower adherence to recommended vaccination schedules, and a higher lost to follow-up rate than an age-matched control group [17]. Adherence to specialty clinic outpatient appointments was addressed in some of the studies as well. In a study assessing ophthalmologic follow-up of infants of opiate-dependent mothers found a poor appointment adherence and a high rate of lost to follow-up [20]. Kivisto et al examined the dental health of opioid-exposed children and found that the exposed group had poorer dental health, a lower likelihood of performing regular dental care at home, lower parental involvement in dental care, lower appointment adherence compared with non-exposed children [15].

**Table 2.** Summary of articles related to healthcare utilization in neonatal abstinence syndrome (NAS) patients after birth hospitalization

Study	Country	Study population	NOS	LOE	Covariates included in multivariate analysis	Insurance breaks or Medicaid coverage	Preventative and specialty care appointment adherence	ER visits	Readmission	Lost to follow-up	Mortality	Key findings
Fang (2015)	TW	Infants born to mothers on methadone maintenance (MM) from population-based databases (*N* = 1056 MM infants, 10,547 normal infants)	8	2b	Maternal factors (insurance, education, marital status, urban residence, prior deliveries, prenatal visits, Child factors (gender, insurance, prematurity)	↓	↓	↑	–	–	–	Infants born to mothers before MM have lower preventative healthcare utilization. Infants born to mothers after MM have more ER visits.
Gill (2003)	AU	Infants born to opiate-dependent mothers at single institution (*N* = 49)	–	4	*Maternal factors and child factors reported but no multivariate analysis performed*	–	↓	–	–	↑	–	Strabismus higher in NAS infants than general population. No control group
Kivisto (2014)	FI	Infants born to women on buprenorphine maintenance (BM) at single institution (*N* = 51 BM, 68 normal infants)	5	3b	*Maternal factors and child factors reported but no multivariate analysis performed*	–	↓	–	–	–	–	Higher incidence of dental caries and neglect in BM infants
Patrick (2015)	US	Infants with NAS diagnosis from population-based database (*N* = 1,643 NAS, 51,748 preterm, 700,613 normal)	7	2b	Maternal factors (insurance type) and child factors (birth weight, respiratory complications, seizures, feeding problems, sepsis, gender, length of stay)	↓	–	–	↑	–	–	NAS infants have a similar readmission rate as preterm infants but higher than uncomplicated term infants
Payot (2000)	CH	Infants with NAS diagnosis at single institution (*N* = 37 children of drug-abusing mothers, 37 controls)	6	3b	*Maternal factors and child factors reported but no multivariate analysis performed*	–	↓	↔	–	↑	–	Children of drug-abusing mothers have longer neonatal hospital stays
Uebel (2015)	AU	Infants with NAS diagnosis from population-based database (*N* = 3,842 NAS infants, 1,018,421 normal infants)	8	2b	Maternal factors (age, rural indigenous, # children, smoking, type of delivery, type of hospital, socioeconomic status) and child factors (gender, rural residence, birth weight, prematurity, APGAR, type of admission)	–	–	–	↑	–	↑	NAS is an independent risk factor for readmission for maltreatment (OR 4.5) and mental/behavioral disorders (OR 2.3)
Witt (2017)	US	Infants with NAS diagnosis from population-based database (*N* = 1,900 NAS infants, 12,283 normal infants)	8	2b	Maternal factors (age, gender, smoking, education, employment, insurance, race, medical complications) and child factors (ICU admission, prematurity, other medical illness)	–	–	–	↑	–	↑	NAS has a higher adjusted relative risk of infectious diseases (1.72), CNS diseases (2.07), asthma (1.82), digestive disease (2.07), GU disease (2.28), skin infections (3.57), neglect/abuse (4.46)

CNS, central nervous system; GU, genitourinary, LOE, Level of Evidence; NOS, Newcastle–Ottawa Scale.

Country codes: AU, Australia, CA, Canada, FI, Finland, CH, Switzerland, TW, Taiwan, US, United States of America.

↑= Increase rates of emergency room (ER) visits, readmission, and mortality in NAS patients compared to controls.

↓= Lower rates of insurance coverage or private insurance, and lower rates of preventative and/or specialty care utilization in NAS patients compared to controls.

↔= No difference in ER visits and lost to follow-up rates between NAS patients and controls.

Readmission rates were higher in NAS patients in three of the included studies. Patrick et al noted increased odds of 30-day readmission following birth hospitalization among both NAS (OR = 2.49) and late preterm infants (OR = 2.26) compared to uncomplicated term infants after adjusting for sex, birth weight, comorbidities, and insurance type [26]. Similarly, Uebel et al demonstrated a higher likelihood of readmission among NAS children (OR = 1.63) along with a higher mortality (OR = 3.25) compared to non-NAS children. This study reported that NAS patients were more likely to be readmitted for respiratory disease, subcutaneous tissue infections, burns, poisoning, maltreatment, accidents, and assault. NAS patients were less likely to be discharged to home following readmission and more likely to be transferred to another hospital [27]. These findings were supported by Witt et al who found that NAS children have higher rates of readmission within the first 5 years of life compared to an unexposed cohort (21.3% vs. 12.7%) [28]. The adjusted relative risk of NAS infant readmission was 1.54 after controlling for maternal age and education, gestational age, and prenatal smoking. The admitting conditions most common among NAS children included parasitic diseases, respiratory conditions, infections, and cellulitis. This study also found a trend toward higher NAS infant mortality.

### Outpatient Weaning Programs

The complex medical and social needs of NAS infants can influence postnatal healthcare utilization. Evaluation of outpatient weaning programs through this review can give important information in gaining a deeper understanding of the needs of these infants and facilitating factors that are necessary to support the health of this very vulnerable population. Seven studies detailed outpatient weaning programs which evaluated patient/caregiver needs assessment, program support resources, and treatment compliance (Table 3). The number of patients treated as outpatients within these programs ranged from 22 to 117. Only two of the studies were not controlled in some fashion [29,30]. These studies reported significant needs of NAS infants within the ambulatory setting. These studies involved assessment of extensive social and medical needs requiring intensive evaluation and follow-up [16,19,29–33]. The complex social and medical needs of these infants required a multidisciplinary collaboration between trained clinical staff, social workers, child welfare agencies [19,29,31]. In order to prevent inappropriate treatment and lapses of treatment, highly knowledgeable outpatient pharmacies had to provide intensive counseling and follow-up through the weaning protocol [30]. Additional supportive measures employed in these programs to prevent non-adherence included home visits, phone calls [32,33], transportation arrangement [32], and 24-hour access to the medical doctor caring for the patients [31]. Finally, parental education on medication administration, weaning protocols, and withdrawal signs and symptoms in the newborn was considered a significant portion in six of the seven programs [19,29–32,33].

**Table 3.** Summary of articles related to outpatient neonatal abstinence syndrome (NAS) weaning programs

Study	*N*	Country	Study population	NOS	LOE	Needs upon enrollment	Support	Compliance	Follow-up
Backes (2012)	46	US	Infants born to mothers on methadone maintenance at single institution	5	3b	Medical, opioid dose, social	Education, 24-hour MD, multidisciplinary clinic	11% ER visits (all NAS); 7% readmitted (all NAS)	None lost to follow-up while on tx; 80% follow-up at 1 year
Chau (2016)	22	US	Infants with NAS diagnosis at single institution	–	4	Medical, opioid dose, social	Education, multidisciplinary clinic	0 ER visits; 3 readmitted (2 NAS)	None lost to follow-up
Dickes (2017)	117	US	Infants born to mothers on methadone or buprenorphine maintenance at single institution	–	4	Medical, social	Education, transportation, home visits	5 oversedated; 14% ER visits; 7% readmitted (0 NAS)	–
Kelly (2015)	52	CA	Infants with NAS diagnosis at two hospitals	6	2b	Medical, opioid dose, social	–	2% ER visit (all NAS); 1 death	–
Lai (2017)	53	US	Infants with NAS diagnosis at single institution	–	4	Medical, social	Education, outpatient pharmacy	5 readmitted (3 NAS)	–
Oei (2001)	26	AU	Infants born to mothers on methadone maintenance at single institution	–	4	Medical, social	Education, multidisciplinary clinic	91.6% clinic attendance	None lost to follow-up
Smirk (2014)	38	AU	Infants with NAS diagnosis at single institution	6	2b	Medical, social, education	Education, home visits, phone calls	2 medication errors; 1 readmitted (1 NAS); 10% of appts. missed	–

NOS, Newcastle–Ottawa Scale; LOE, Level of Evidence; (# NAS), Indicates number or rate of NAS symptom-related healthcare interventions (e.g. ER visits, readmissions); Sx, symptoms; Tx, treatment; Appts, appointments.

Country codes: AU, Australia, CA, Canada, FI, Finland, CH, Switzerland, TW, Taiwan, US, United States of America.

With the extensive social and medical support described above, these studies reported the positive effects of aggressive outpatient support programs in this population. Treatment compliance was mostly measured by clinic attendance, ER visits, and hospital readmissions due to withdrawal symptoms. Two studies reported clinic attendance of 90% and 92% for all scheduled visits among all patients [19,33]. Four studies included ER visits due to NAS symptoms in their data ranging from 0 to 11% of patients [16,29,31,32]. Readmission rates were examined in five studies ranging from 0 to 9% [29–32,33]. Smirk et al discussed two medication errors (one infant was inappropriately weaned at the parent’s discretion, another was given morphine at home after completion of therapy due to perceived NAS symptoms) [33]. Four percent of patients in the program described by Dickes, et al. experienced oversedation [32]. One death was described which was a case of sudden infant death syndrome in a child undergoing at-home oral morphine weaning [16]. Treatment follow-up was excellent among those studies reporting outcomes. Three studies cited zero patients being lost to follow-up while undergoing treatment [19,29,31]. Understanding the success of these weaning programs and the inherent complexity of this pediatric population provides insights on intensive support and resources for this population that could be applied in other aspects of health to promote appropriate healthcare utilization of primary and specialty care throughout childhood.

## Discussion

The social environment surrounding many children with NAS is plagued with lower levels of parental education, lower socioeconomic status, and inconsistent health insurance coverage [6,7]. Furthermore, increased rates of placement into foster care or adoption services, involvement of child protective services, and social situation involving parental abuse among this population contribute to the chaotic surroundings [8–11]. The epidemiological expansion of NAS throughout resource-limited regions, such as rural areas, increases the complexity of providing comprehensive care for these children [7,12]. These factors can negatively affect NAS infants and utilization of healthcare [18,22,34–37] Four of the utilization studies employed multivariate analysis to control for maternal and child factors that could influence result [25–28]. These studies have reported that NAS represents an independent overall health and/or healthcare utilization risk factor. This review has found that NAS patients have poorer utilization of preventative and specialty healthcare services than unexposed controls once discharged from their birth hospitalization. Insurance coverage, among other sociodemographic factors, may play a role in decreased utilization; however, there remains a gap in our understanding of the key factors influencing utilization. The overall health of these infants is poorer as indicated by the increased ER visits, hospital readmissions, and overall mortality seen in several of the studies represented. These findings are congruent with those seen in patients with other chronic health conditions or younger children from lower income families [38,39]. It is difficult to draw conclusions from some of the studies that did not include adequate controls or multivariate analysis [15,17,20]. Furthermore, diverse reporting of types of healthcare utilization outcomes as well as limited reporting timing of utilization prevented meta-analysis.

In spite of the poor determinants of health of the NAS population, infants enrolled in outpatient weaning programs displayed excellent rates of compliance, utilization of services, and follow-up. This provides important insights that could be applied to other areas of outpatient healthcare to support more appropriate utilization of services to promote overall health of NAS infants. The success of these programs may be connected to the intensive support mechanisms in place. These studies do contain inherent selection bias, as the families eligible for outpatient services underwent extensive screening prior to enrollment. One study reported that, compared to an inpatient weaning program group, the outpatient group had decreased odds of child protective services involvement once removed from the hospital [33]. A deeper understanding of factors that influence health of this population is valuable to the healthcare system. The practice of discharging patients from the hospital prior to completion of weaning is not unique to the studies included here. According to a 2009 survey from Ireland and the UK, 29% of 211 responding neonatal units practiced outpatient weaning [40]. There is increasing use of outpatient support, such as weaning programs, for NAS children and some centers are reporting significant cost savings to the healthcare system (approximately $93,400) [1]. Before considering the overall cost benefit from these programs, it is critical to gain a deeper understanding on how who best be served by outpatient programs which is an area that neither the overall literature nor this review definitively address. There is a definite need to determine very clear evidence-based criteria for the type of intervention that NAS infants/families should be enrolled in. Increased access to psychosocial support and care navigation services may improve some utilization but may come at a greater cost. Further studies to compare costs between inpatient and outpatient management of NAS, with consideration of the resources necessary in each setting, could help guide the most responsible treatment of these children. Considering the complexity of NAS patients, it is clear that these patients require extensive discharge planning and outpatient care coordination. Those who have completed inpatient weaning require education in withdrawal signs, social support, adequate posthospital follow-up, and rehabilitative support should drug abuse remain a concern. Within the USA, NAS infants can receive resources and services that are provided through legislation of the Child Abuse Prevention and Treatment Act [21]. The evidence that NAS infants are at higher risk for neglect and/or abuse could potentially be utilized by state and local agencies to mobilize these resources efficiently beyond the hospital discharge. Future work is needed to better understand the needs of NAS children and translate benefits identified from successful weaning programs into other healthcare disciplines.

There are multiple limitations of this review as well as the articles included in the review. Many of the studies have small sample sizes and lack of complete data regarding timing of infant healthcare utilization complicates the interpretation of the findings of some of the studies. In spite of thorough search criteria, it is possible that relevant articles were excluded from this review. Outcome reporting bias is also a limitation of these studies based on the broad variety of measures and methods of data collection. There is significant bias within these articles due to the nature of the study design of most of the articles (cohort, case-control, and case series designs). Many of the studies represented pilot work with small sample sizes (< 50), which could be a poor representation of the overall population, which introduced selection bias and publication bias. Furthermore, there was a wide age difference within some studies with limited analysis to control for sociodemographic and/or economic factors. Conclusions drawn from this systematic review are limited, as a meta-analysis could not be performed.

## Conclusion

Regarding healthcare utilization and access following the birth hospitalization, NAS children have poorer outpatient appointment adherence and have a higher rate of being lost to follow-up. These children have overall poorer health indicated by a significantly higher risk of ER visits, hospital readmission, and early childhood mortality compared with non-NAS infants. The intensive multidisciplinary support provided through outpatient weaning programs serves as a model that could be applied to other healthcare fields to support over better health as well as improved healthcare utilization among this population.
